# Supplementary material for: Phage diversity mirrors bacterial strain diversity in the honey bee gut microbiota
Source: Nat Commun. 2025 Nov 4;16:9738. doi: 10.1038/s41467-025-64706-2 (PMC12586679; doi:10.1038/s41467-025-64706-2)
Supplement: Supplementary file 1 — Supplementary Information [file 41467_2025_64706_MOESM1_ESM.pdf]

Supplementary Information for

Phage diversity mirrors bacterial strain diversity in the honey bee gut microbiota

Malick Ndiaye, Germán Bonilla-Rosso, Florent Mazel, Philipp Engel*

*To whom correspondence should be addressed

e-mail: philipp.engel@unil.ch

Supplementary Figures

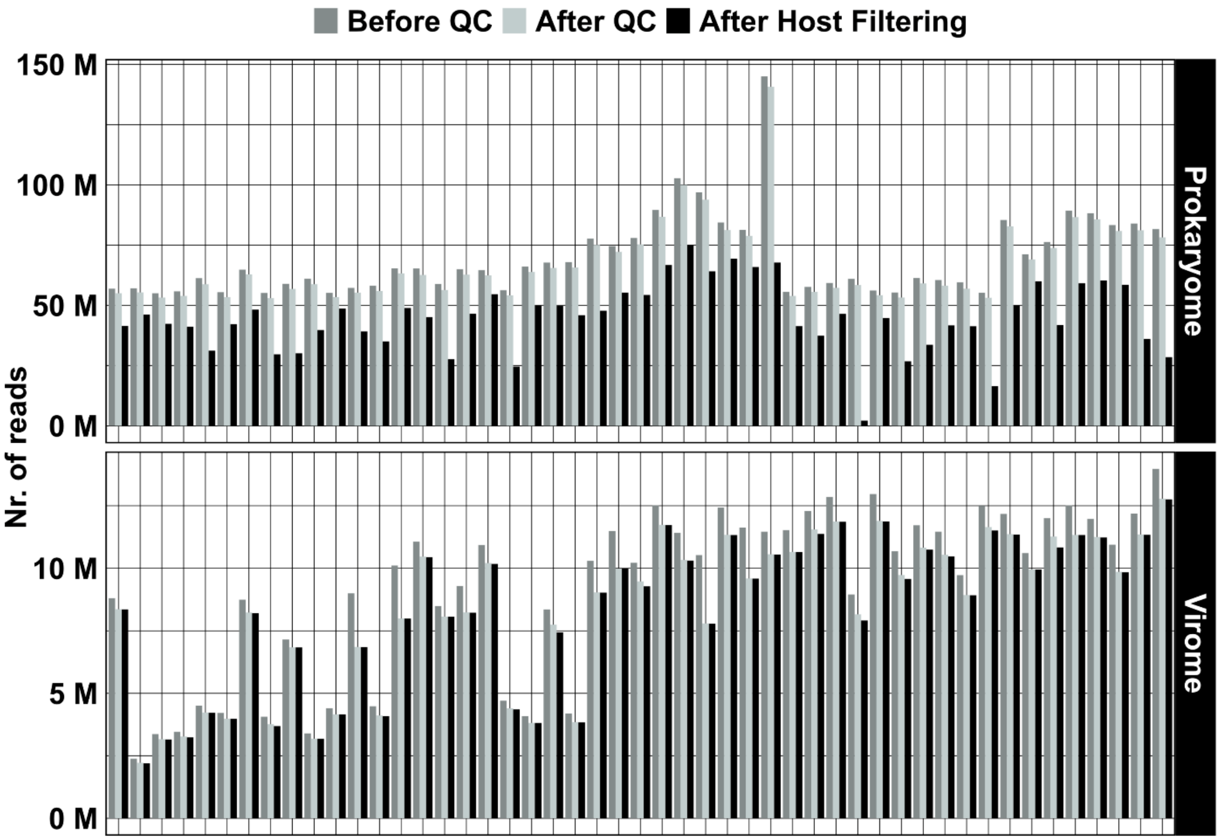

Supplementary Figure 1: Shotgun metagenomics sequencing of the viral and bacterial fraction of 49 gut microbiomes of individual honeybees. Number of reads obtained for the bacterial (top) and viral (bottom) fraction of the 49 samples after sequencing, quality-check, and filtering for reads belonging to the honeybee genome.

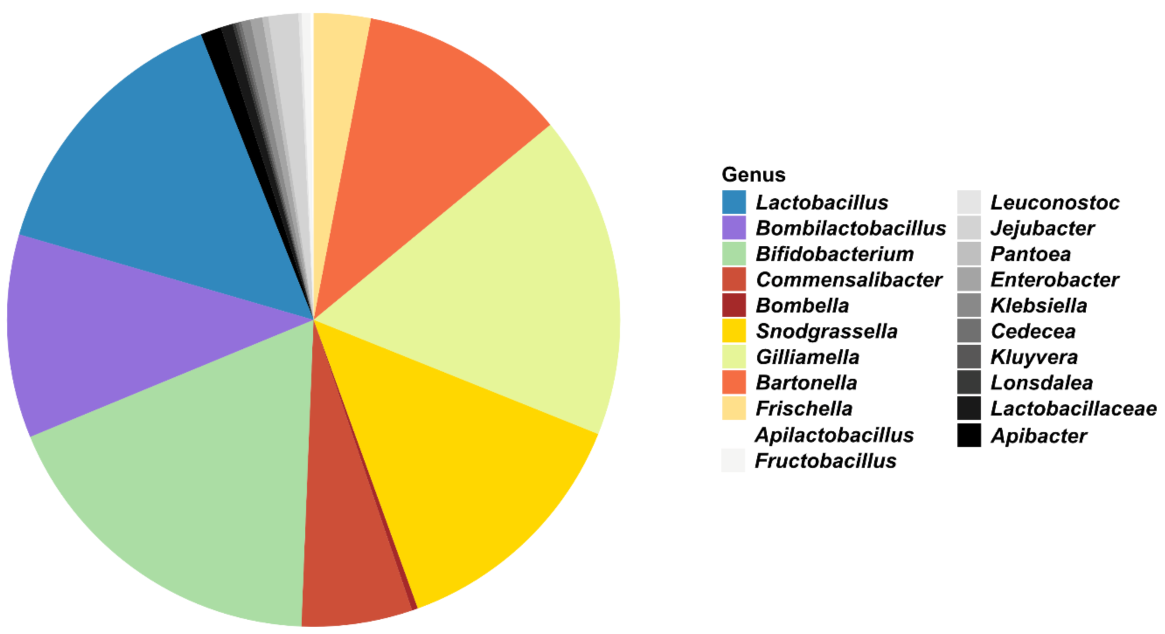

Supplementary Figure 2: bMAGs recovered from the 49 honeybee bacterial metagenomes. Pie chart illustrating the proportions of bMAGs obtained from 49 honeybee bacterial metagenomes that belong to different genera. Each slice of the pie corresponds to a specific genus, with honeybee-associated genera highlighted in distinct colors, while other genera are represented in varying shades of grey.

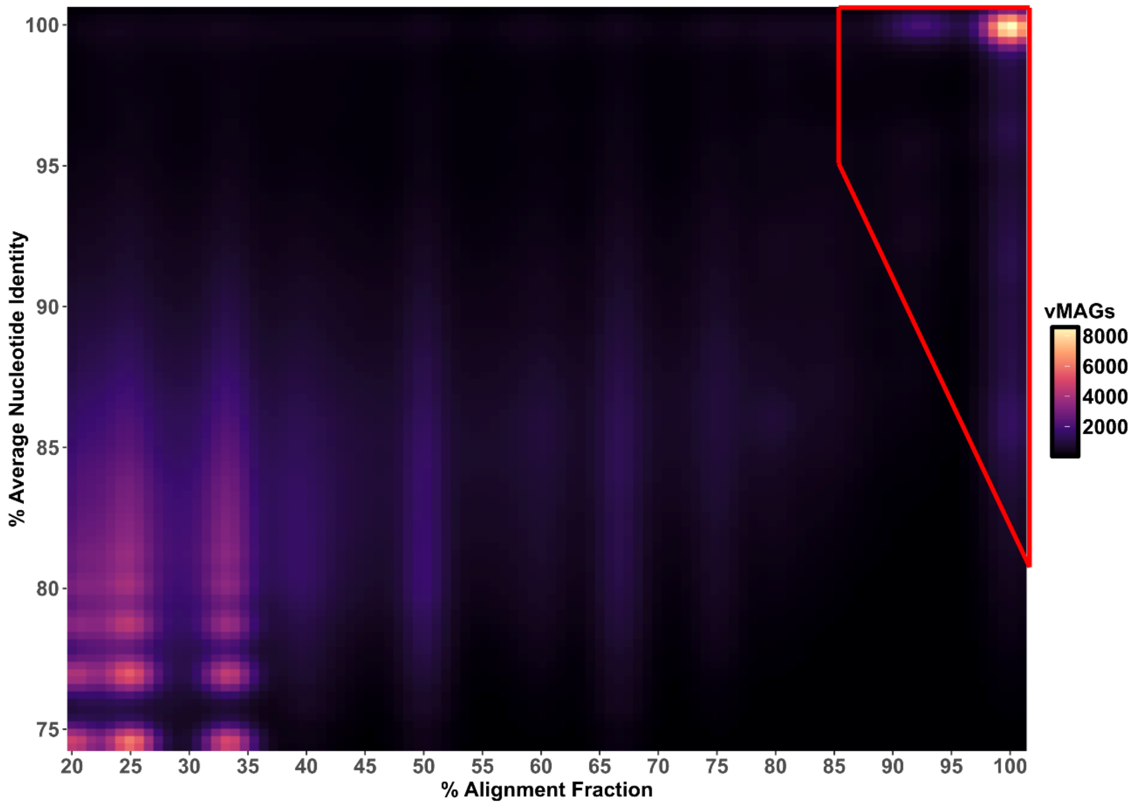

Supplementary Figure 3: Pairwise differences between vMAGs used to cluster vOTU. Number of vMAG pairs displaying a given average nucleotide identity (ANI) and alignment fraction (AF). The red perimeter indicates vMAGs pairs with whole genome ANI (ANI*AF) >= 0.8, and considered in this study to belong to the same vOTU.

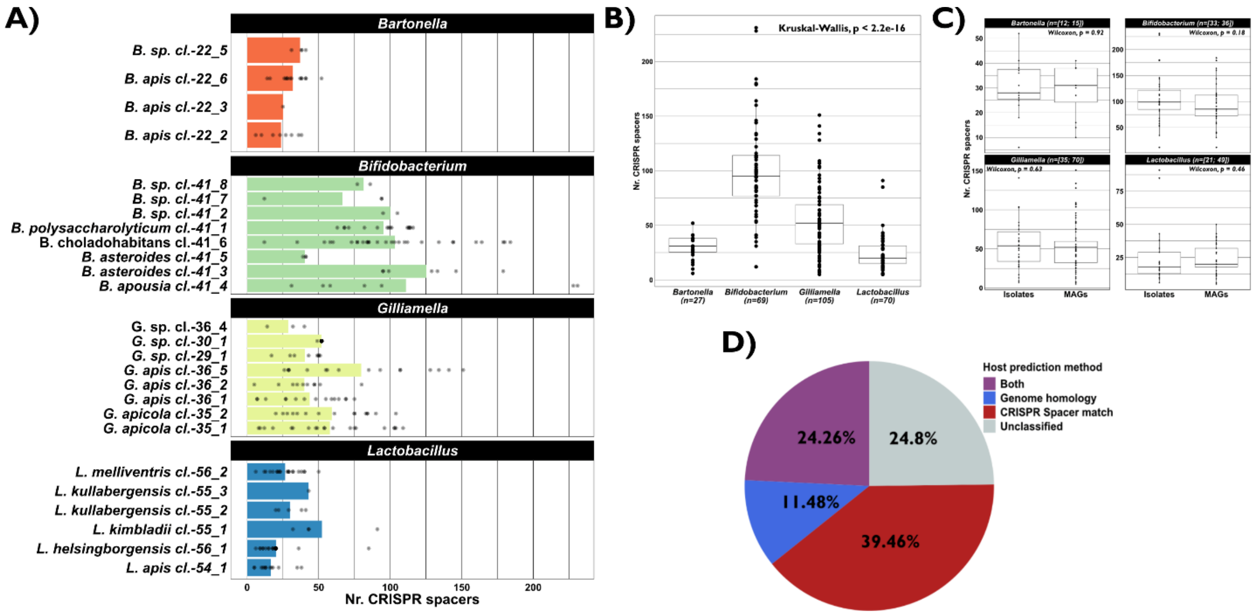

Supplementary Figure 4: Analysis of CRISPR spacers recovered from the bacterial genomes. (A) Barplot illustrating the number of spacers recovered from the genomes of each bOTU (species). Each row in the plot corresponds to a specific bOTU, and colors represent the genus of the bOTU. (B) Boxplot displaying the distribution of the number of CRISPR spacers recovered in genomes, categorized by genus. The Kruskal-Wallis p-value, indicating significant differences among genera, is presented on the plot. (C) Boxplots comparing the distribution of the number of CRISPR spacers identified in bMAGs, versus isolated genomes for each genus. The Wilcoxon p-value for the comparison is provided on the plots. (D) Pie chart illustrating the method of host prediction for the vMAGs of medium- to complete quality. If a vMAG was detected by spacer-to-protospacer match and by genome homology, it is classified under both.

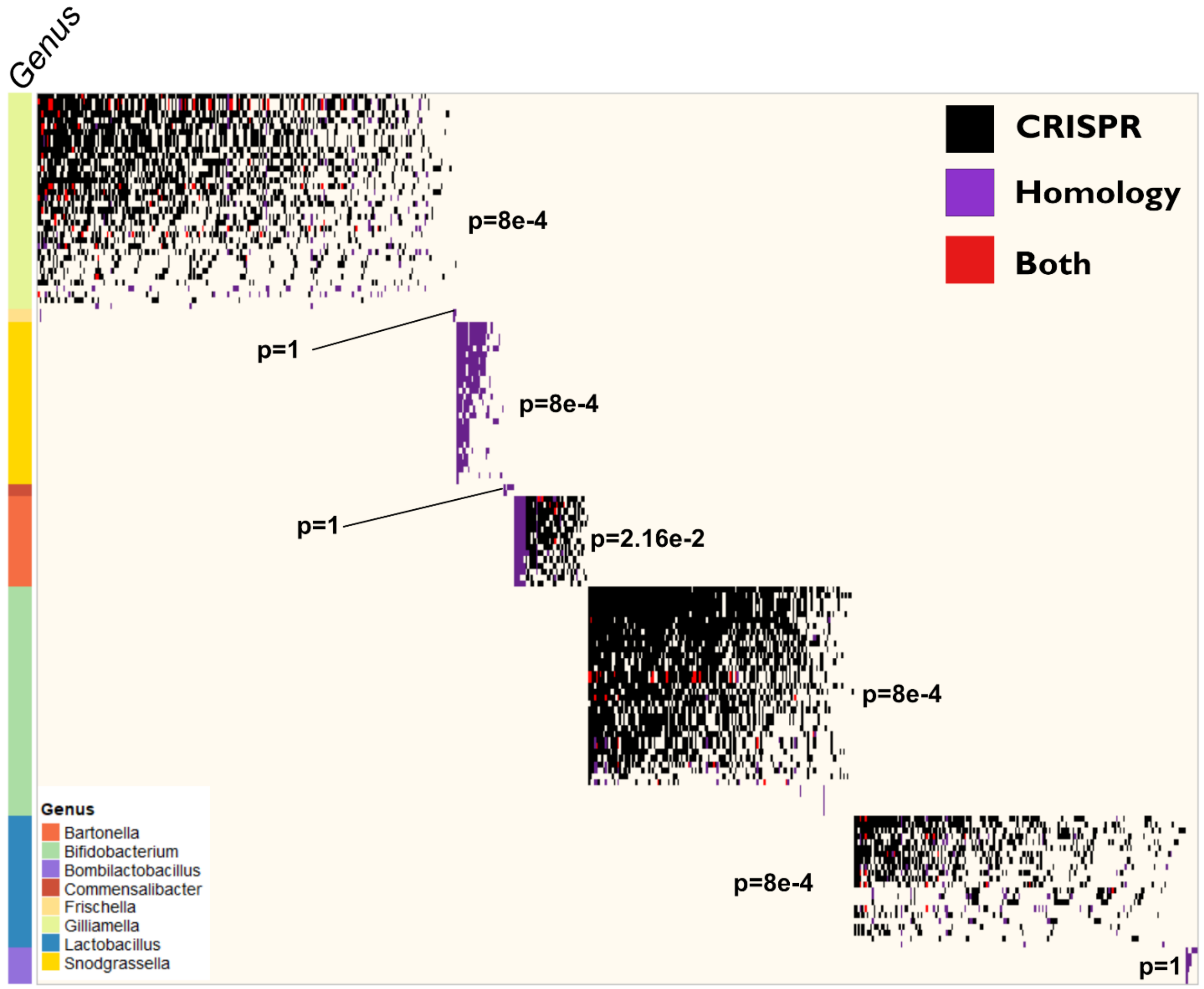

Supplementary Figure 5: Phage-bacteria interaction network (PBIN) based on isolate bacterial genomes. Predicted phage bacteria interactions using only isolate bacterial genomes and vOTUs are shown by colored dots. Each interaction is represented by a colored dot, where the dot's color indicates the method used to predict the phage-host linkage. The genus of each bacterial genome is denoted by colored bars adjacent to the rows of the network matrices. Bonferroni adjusted p-value of nestedness within each module (estimated using null models, see methods) is shown next to the respective module in the matrix.

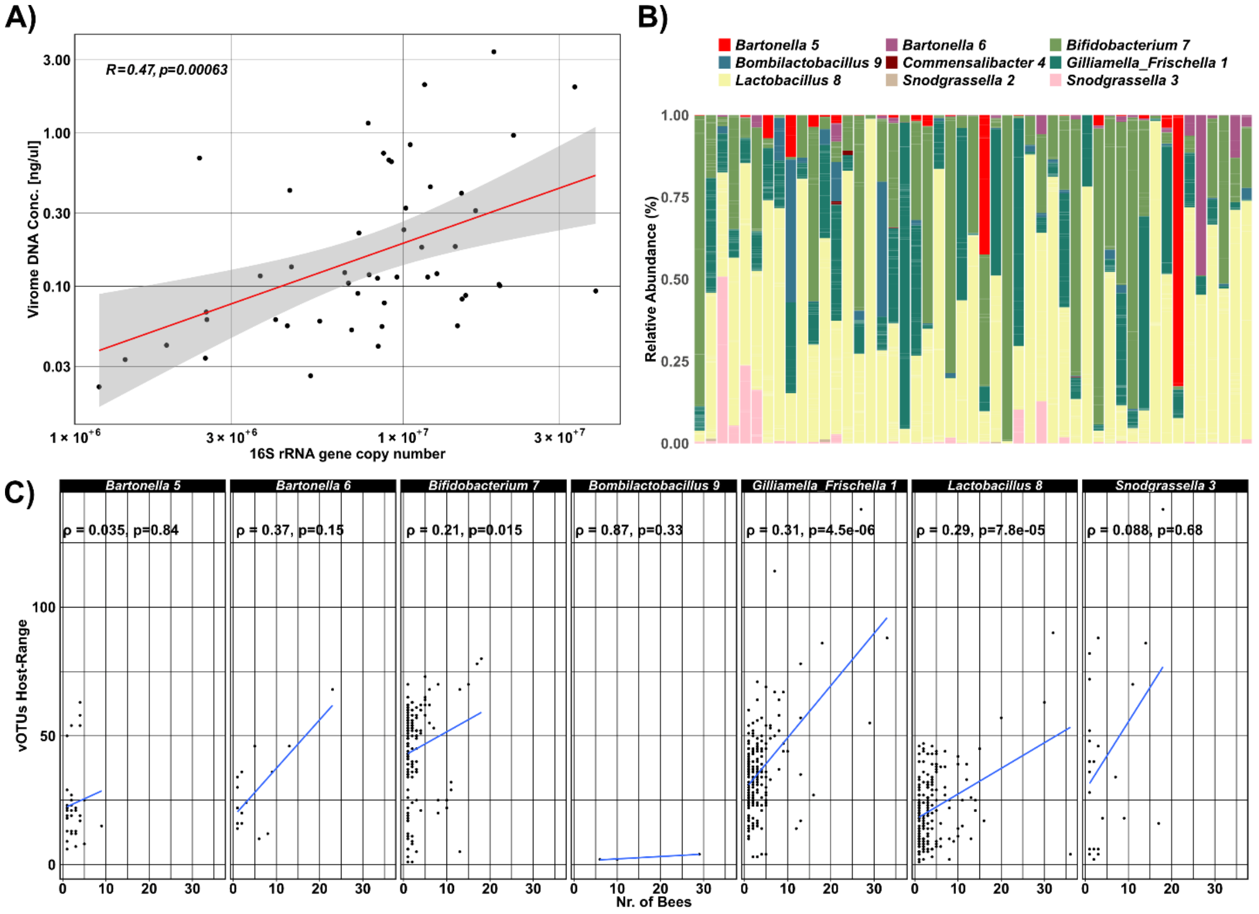

Supplementary Figure 6: Overview of viral community estimated biomass, composition and distribution in 49 individual honeybees. (A) Correlation between the 16S rRNA gene copy number in the bacterial fraction (x-axis) and DNA yield from the virome fraction (y-axis). (B) Community composition of viruses assigned to an Interaction module. Colors represent different interaction modules. (C) Correlation between vOTU prevalence across individual bees and host range, shown separately for each interaction module (IM). Host range is defined as the number of bacterial genomes with which a given vOTU was predicted to interact (Fig. 2B). Spearman's rho and p-values are indicated on each plot, along with a blue regression line for visual reference. Only IMs containing both phages and bacteria detected in at least 15 bees were included in this analysis.

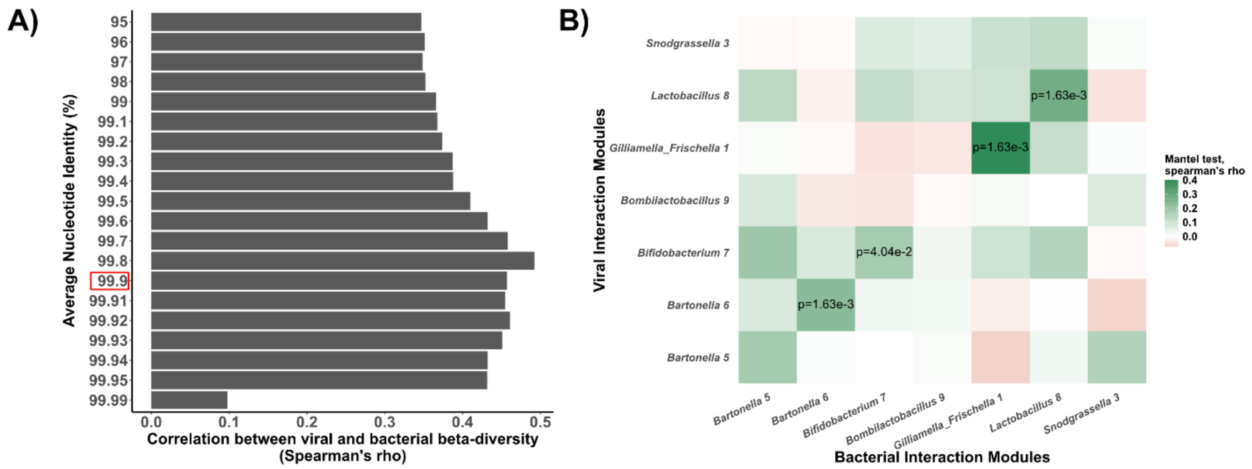

Supplementary Figure 7: Break-down of beta-diversity correlations between viral and bacterial community. (A) Correlation coefficients (Spearman's rho) between viral Jaccard compositional dissimilarity and bacterial Jaccard compositional dissimilarity across a range of ANI thresholds used to define strain-sharedness, from 95% (species level) to 99.99% ANI. The selected threshold of 99.9% ANI, used for strain-sharedness analyses in this study, is highlighted with a red box. (B) Heatmap displaying mantel test results (Spearman's rho) obtained by comparing the vOTU Jaccard dissimilarity matrix with the bacteria strain-level Jaccard dissimilarity (99.9% ANI) matrix, stratified by interaction module. Only interaction modules with phages and bacteria detected in at least 15 bees were tested. P-values for correlations between IMs of viral and bacterial fractions, assessed using the Mantel test (1000 permutations) and adjusted for multiple comparisons with the *fdr* method, are reported only when $p < 0.05$.

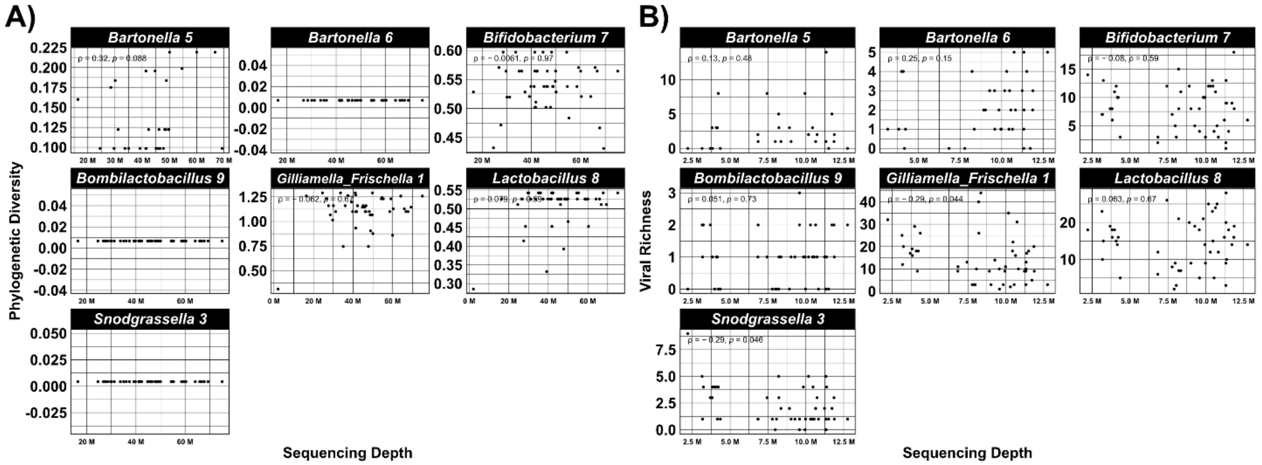

Supplementary Figure 8: Relationship between sequencing depth and observed diversity. The plots are categorized by interaction module. Spearman's rho correlation between bacterial or viral richness and sequencing depth are presented along with their associated p-value are displayed on each plot. (A) Scatter plot illustrating the relationship between the bOTU phylogenetic diversity and sequencing depth. (B) Scatter plot illustrating the relationship between the vOTU richness and sequencing depth.

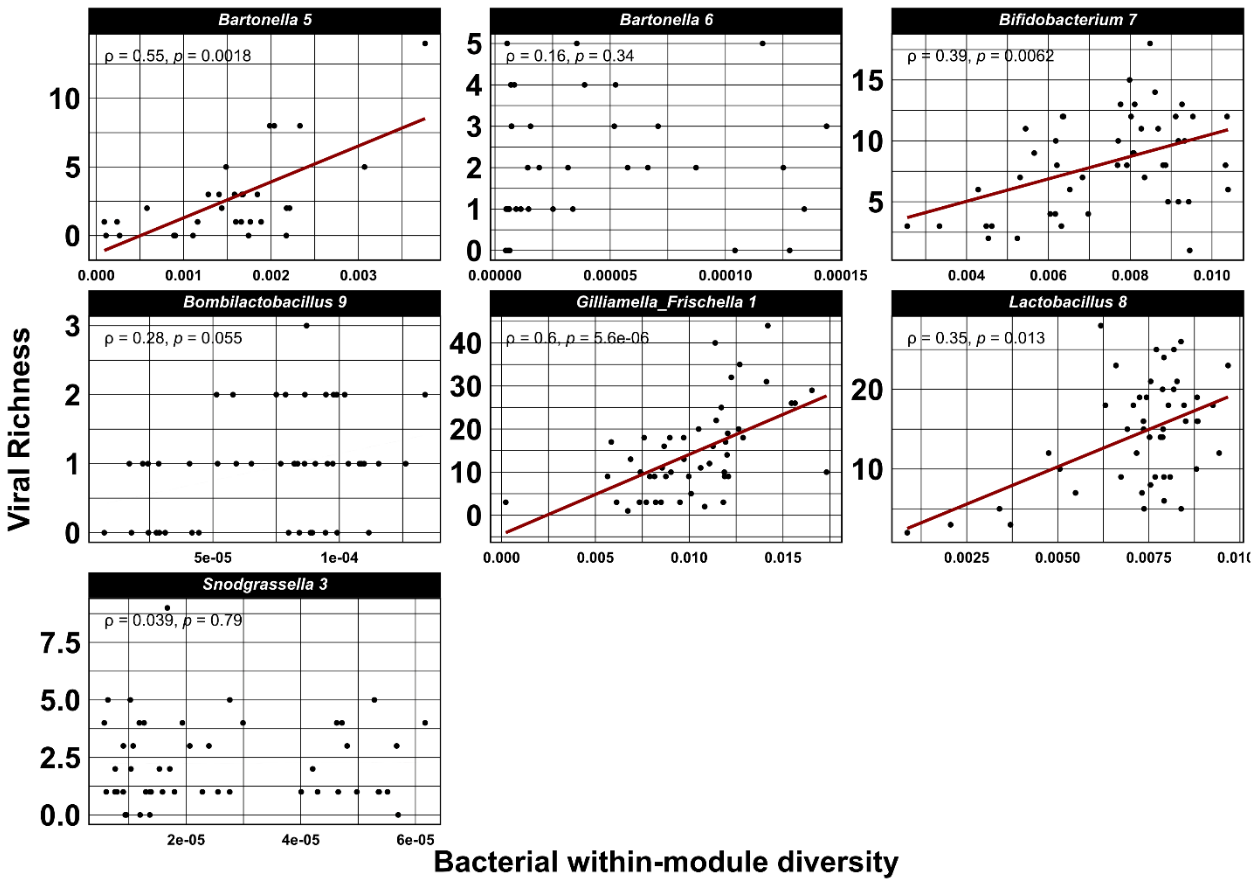

Supplementary Figure 9: Relationship between viral and bacterial alpha diversity within each individual module. Correlation between bacterial within-module diversity (phylogenetic diversity × average nucleotide diversity) and viral richness (vOTUs count). Each panel corresponds to a distinct interaction module. Spearman's rho and p-values are shown on the plot, with a red linear regression line indicating significant correlations.

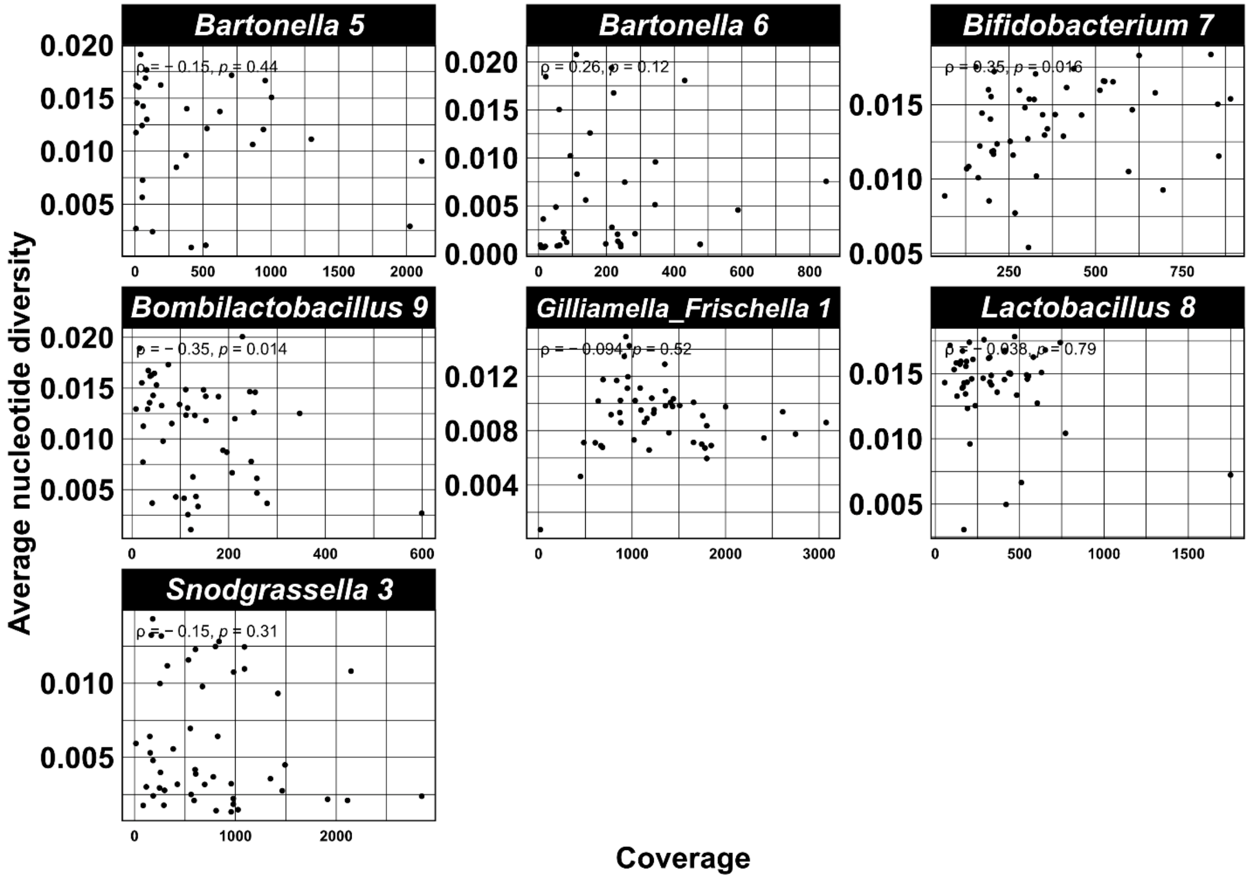

Supplementary Figure 10: Relationship between coverage and bacterial average nucleotide diversity. Scatter plots illustrating the relationship between the average nucleotide diversity and sequencing depth. The plots are categorized by interaction module. Spearman's rho and p-value are displayed on each plot.

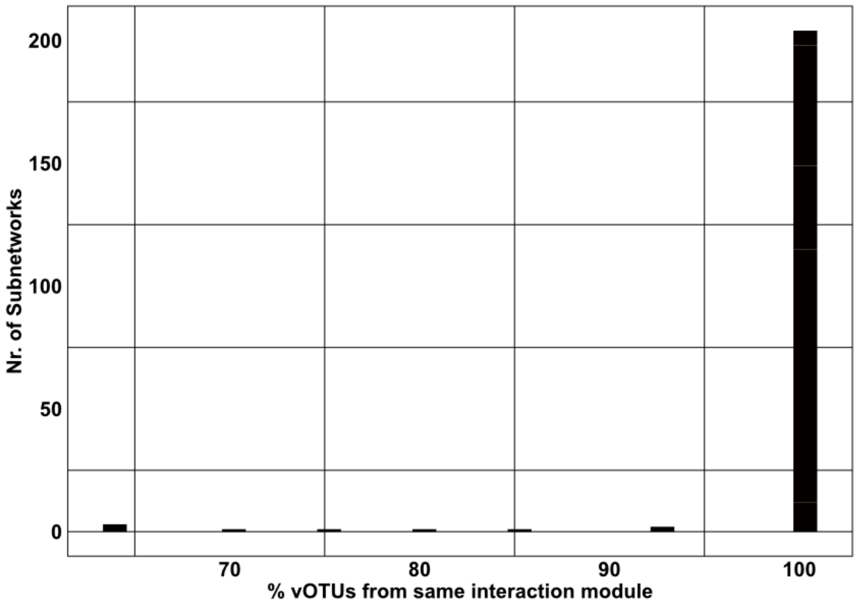

Supplementary Figure 11: Clustering of vOTU according to interaction module (IM) in proteome-sharedness network. Histogram showing the percentage of vOTUs in local subnetworks belonging to the same interaction module. 96% of local subnetwork are composed exclusively of vOTUs from the same interaction module.

Supplementary Data

Supplementary Data 1. (separate file: Supplementary-Data-1.xlsx)

Sample Information and collection Metadata.

Supplementary Data 2. (separate file: Supplementary-Data-2.xlsx)

Information on reads and bases retained after read quality filtering (PHREAD score >= 28) and host reads filtering.

Supplementary Data 3. (separate file: Supplementary-Data-3.xlsx)

Information on bacterial genomes (isolate genomes and Metagenome-assembled genomes)

Supplementary Data 4. (separate file: Supplementary-Data-4.xlsx)

Information on viral MAGs

Supplementary Data 5. (separate file: Supplementary-Data-5.xlsx)

Information on CRIPR spacers recovered from bacteral MAGs and isolate genomes.

Supplementary Data 6. (separate file: Supplementary-Data-6.xlsx)

Phage-host linkage information

Supplementary Data 7. (separate file: Supplementary-Data-7.xlsx)

Between and within interaction module beta-diversity mantel test results.

Supplementary Data 8. (separate file: Supplementary-Data-8.xlsx)

Correlations between viral richness and bacterial nucleotide diversity, within and across interaction modules (Spearman's correlation).

Supplementary Data 9. (separate file: Supplementary-Data-9.xlsx)

Information on isolate genomes

Supplementary Data 10. (separate file: Supplementary-Data-10.xlsx)

Beta-diversity mantel test results between viral and bacterial community at different popANI threshold to define bacterial strain-sharedness.
