# Supplementary material for: Phage diversity mirrors bacterial strain diversity in the honey bee gut microbiota
Source: Nat Commun. 2025 Nov 4;16:9738. doi: 10.1038/s41467-025-64706-2 (PMC12586679; doi:10.1038/s41467-025-64706-2)
Supplement: Supplementary file 2 — Description of Additional Supplementary Files [file 41467_2025_64706_MOESM2_ESM.pdf]

Title: Supplementary Data 1

Description: Sample Information and collection Metadata.

Title: Supplementary Data 2

Description: Information on reads and bases retained after read quality filtering (PHREAD score >= 28) and host reads filtering.

Title: Supplementary Data 3

Description: Information on bacterial genomes (isolate genomes and Metagenome-assembled genomes)

Title: Supplementary Data 4

Description: Information on viral MAGs

Title: Supplementary Data 5

Description: Information on CRIPR spacers recovered from bacteral MAGs and isolate genomes.

Title: Supplementary Data 6

Description: Phage-host linkage information

Title: Supplementary Data 7

Description: Between and within interaction module beta-diversity mantel test results.

Title: Supplementary Data 8

Description: Correlations between viral richness and bacterial nucleotide diversity, within and across interaction modules (Spearman's correlation).

Title: Supplementary Data 9

Description: Information on isolate genomes

Title: Supplementary Data 10

Description: Beta-diversity mantel test results between viral and bacterial community at different popANI threshold to define bacterial strain-sharedness.
