# Supplementary material for: Phage diversity mirrors bacterial strain diversity in the honey bee gut microbiota
Source: Nat Commun. 2025 Nov 4;16:9738. doi: 10.1038/s41467-025-64706-2 (PMC12586679; doi:10.1038/s41467-025-64706-2)
Supplement: Supplementary file 14 — Reporting Summary [file 41467_2025_64706_MOESM14_ESM.pdf]

nature portfolio

Corresponding author(s): Philipp Engel
Last updated by author(s): Malick Ndiaye

Reporting Summary

Nature Portfolio wishes to improve the reproducibility of the work that we publish. This form provides structure for consistency and transparency in reporting. For further information on Nature Portfolio policies, see our Editorial Policies and the Editorial Policy Checklist.

Statistics

For all statistical analyses, confirm that the following items are present in the figure legend, table legend, main text, or Methods section.

n/a	Confirmed	
☐	☒	The exact sample size (*n*) for each experimental group/condition, given as a discrete number and unit of measurement
☐	☒	A statement on whether measurements were taken from distinct samples or whether the same sample was measured repeatedly
☐	☒	The statistical test(s) used AND whether they are one- or two-sided<br>*Only common tests should be described solely by name; describe more complex techniques in the Methods section.*
☐	☒	A description of all covariates tested
☐	☒	A description of any assumptions or corrections, such as tests of normality and adjustment for multiple comparisons
☐	☒	A full description of the statistical parameters including central tendency (e.g. means) or other basic estimates (e.g. regression coefficient) AND variation (e.g. standard deviation) or associated estimates of uncertainty (e.g. confidence intervals)
☐	☒	For null hypothesis testing, the test statistic (e.g. F, t, r) with confidence intervals, effect sizes, degrees of freedom and P value noted<br>*Give P values as exact values whenever suitable.*
☒	☐	For Bayesian analysis, information on the choice of priors and Markov chain Monte Carlo settings
☒	☐	For hierarchical and complex designs, identification of the appropriate level for tests and full reporting of outcomes
☐	☒	Estimates of effect sizes (e.g. Cohen's *d*, Pearson's *r*), indicating how they were calculated

Our web collection on statistics for biologists contains articles on many of the points above.

Software and code

Policy information about availability of computer code

Data collection: No software has been used for data collection. all data used was producted in this study

Data analysis: Code and details of parameters and software used are available at https://github.com/MalickNdiye/PHOSTER archived at https://doi.org/10.5281/zenodo.17087213.

Third party softawers used for data analysis in this study:
- Trimmomatic v0.35
- bbsplit v38.18
- Kraken2 v2*
- Filtlong v0.2.1
- Flye v2.9.1
- GraphMap v0.5.2
- Racon v1.5.0
- Bowtie2 v2.4.2
- Pilon v1.22
- MetaSpades v3.15
- metabat2 v2.15
- CheckM v1.0.13
- dRep v3.4.0
- GTDB-Tk v2.1.1

- MetaViralSpades v3.15
- VIBRANT v1.2.1
- Virsorter2 v2.2.3
- viralVerify v1.1
- FastANI v1.33
- CheckV v1.0.1
- CRISPRCasFinder v4.2.20
- DefenseFinder v1.1.1
- OrthoFinder v2.2.7
- prodigal v2.6.3
- IQtree v2.3.6
- vConTACT v2.0
- Bowtie2 v2.5.1
- inStrain v2.5.1
- r-picante v1.8.2
- r-vegan v2.6-4
- r-lpbrim v1
- r-ggpubr v0.6.0
- r-MASS v7.3-60
- r-lmtest v0.9_4

For manuscripts utilizing custom algorithms or software that are central to the research but not yet described in published literature, software must be made available to editors and reviewers. We strongly encourage code deposition in a community repository (e.g. GitHub). See the Nature Portfolio guidelines for submitting code & software for further information.

Data

Policy information about availability of data

All manuscripts must include a data availability statement. This statement should provide the following information, where applicable:

- Accession codes, unique identifiers, or web links for publicly available datasets
- A description of any restrictions on data availability
- For clinical datasets or third party data, please ensure that the statement adheres to our policy

Raw metagenomic data has been deposited to the NCBI Sequence Read Archive (SRA) under the Project ID PRJNA1232403 [https://www.ncbi.nlm.nih.gov/bioproject/?term=PRJNA1232403]. Newly sequenced bacterial genomes have been deposited to the NCBI genome portal under the Project ID PRJNA1232403 [https://www.ncbi.nlm.nih.gov/bioproject/?term=PRJNA1232403] as the following BioSamples: SAMN49894004 [https://www.ncbi.nlm.nih.gov/biosample/?term=SAMN49894004], SAMN49894003 [https://www.ncbi.nlm.nih.gov/biosample/?term=SAMN49894003], SAMN49894002 [https://www.ncbi.nlm.nih.gov/biosample/?term=SAMN49894002], SAMN49894001 [https://www.ncbi.nlm.nih.gov/biosample/?term=SAMN49894001], SAMN49894000 [https://www.ncbi.nlm.nih.gov/biosample/?term=SAMN49894000], SAMN49893999 [https://www.ncbi.nlm.nih.gov/biosample/?term=SAMN49893999], SAMN49893998 [https://www.ncbi.nlm.nih.gov/biosample/?term=SAMN49893998], SAMN49893997 [https://www.ncbi.nlm.nih.gov/biosample/?term=SAMN49893997], SAMN49893996 [https://www.ncbi.nlm.nih.gov/biosample/?term=SAMN49893996]. The collection of bacterial metagenome-assembled genomes (bMAGs), isolate genomes, viral MAGs (vMAGs), as well as scripts, intermediate data files, and tables used to generate the figures in this manuscript are available in the Zenodo repository https://doi.org/10.5281/zenodo.16744037.

Research involving human participants, their data, or biological material

Policy information about studies with human participants or human data. See also policy information about sex, gender (identity/presentation), and sexual orientation and race, ethnicity and racism.

Reporting on sex and gender	n/a
Reporting on race, ethnicity, or other socially relevant groupings	n/a
Population characteristics	n/a
Recruitment	n/a
Ethics oversight	n/a

Note that full information on the approval of the study protocol must also be provided in the manuscript.

Field-specific reporting

Please select the one below that is the best fit for your research. If you are not sure, read the appropriate sections before making your selection.

☐ Life sciences ☐ Behavioural & social sciences ☒ Ecological, evolutionary & environmental sciences

For a reference copy of the document with all sections, see nature.com/documents/nr-reporting-summary-flat.pdf

Ecological, evolutionary & environmental sciences study design

All studies must disclose on these points even when the disclosure is negative.

Study description

Study on gut microbiota (virome and prokaryome) of Apis mellifera.

Research sample

Hindguts of individual Apis mellifera workers were separated into viral and bacterial fractions. The workers were sampled from within the colony and were therefore likely to belong to the nurse caste, although this was not confirmed with additional analyses.

Sampling strategy

Bees were collected from two colonies within the same apiary in Lausanne, Switzerland, with 22 individuals sampled from one colony and 27 from the other (n = 49). Our aim was to obtain at least 20 bees per colony; however, no power analysis was performed, as no comparable studies were available at the time of sampling to estimate an expected effect size. Sampling two colonies was intended to ensure that our results were not colony-specific.

Data collection

We collected 49 adult female worker bees of A. mellifera from 2 hives in Lausanne (Switzerland) in spring 2021. The bees were anesthetized using CO2 and put on ice. Then, they were individually dissected to extract the entire hindgut (pylorus, ileum, rectum). Each hindgut was placed in a tube containing beads (0.75-1 mm glass beads; Carl Roth) and sterile SM buffer (200 mM NaCl, 10 mM MgSO4, 50 mM Tris-HCl pH 7.5, 0.01% gelatin). Samples were homogenized at 6 m/s for 40 sec in a Fast-Prep24 5G homogenizer (MP Biomedicals). Following centrifugation (17'000g for 3min at 4°C), the pellet was stored at -20°C for later DNA extraction of the bacterial fraction. The supernatant was recovered and sequentially filtered through a 0.45m cellulose acetate filter and a 0.22m centrifuge tube filter (Corning Costar Spin-x). Then, the filtrate was treated with DNase I and RNase A (Sigma-Aldrich) for 1h30min at 37°C to degrade free nucleic acids not protected by capsids. The nucleases were inhibited using a lysis buffer (EDTA 0.5mM, Tris 1M and ddH2O) and incubation at 65°C for 10min. Next, VLPs were lysed by one incubation with 75L SDS 10% and 12.5L proteinase K (20mg/mL).

The pellets from the bacterial fraction were thawed at room temperature. Then, bacterial cells were resuspended in 1X PBS, 2X CTAB, 2L ß-mercaptoethanol and 20L proteinase K (20mg/mL). Next, 0.1mm Zirkonia/Silica beads were added to the solution and cells were lysed at 6 m/s for 40 sec in a Fast-Prep24 5G homogenizer (MP Biomedicals) followed by incubation at 56°C for 1h.

Both DNA from the bacterial and phage fractions was extracted with a phenol/chloroform/isoamyl alcohol protocol (Sigma-Aldrich), followed by precipitation with 70% ETOH and linear polyacrylamide (Sigma-Aldrich) overnight, as well as washings with EtOH. The DNA was finally eluted in nuclease-free water and submitted to a further clean up using DNA-specific magnetic beads (clean NGS). DNA concentration was assessed using Qbit.

Libraries were prepared with the Illumina Nextera Flex library kit (Illumina) with unique dual indices (UDI) and sequenced on an Illumina NovaSeq 6000 instrument (PE150) at the Genomic Technologies Facility (GTF) of the University of Lausanne. The quality of the reads was assessed with FastQC (v0.11.4, Babraham Institute) and subsequently trimmed and filtered to remove low quality sequences and short reads using the tool Trimmomatic v0.3571 with settings PE ILLUMINACLIP:NexteraPE-PE.fa:2:30:10 LEADING:28 TRAILING:28 MINLEN:40. Following low-quality reads removal, reads mapping to the A. mellifera and the human genome were removed using bbsplit v38.1872.

The entire data collection procedure was performed by Malick Ndiaye.

Timing and spatial scale

All samples were collected at the University of Lausanne (Switzerland) between the 25.05.2022 and the 20.07.2022

Data exclusions

No samples were excluded from the analyses

Reproducibility

Illumina libraries are stored at -80°C. Code of the entire analysis is available and will allow to reproduce the results based on the same samples.

Randomization

Bees were randomly collected from a given colony.

Blinding

The samples were not blinded during DNA extraction as samples were processed immediately after collection.

Did the study involve field work? ☐ Yes ☒ No

Reporting for specific materials, systems and methods

We require information from authors about some types of materials, experimental systems and methods used in many studies. Here, indicate whether each material, system or method listed is relevant to your study. If you are not sure if a list item applies to your research, read the appropriate section before selecting a response.

Materials & experimental systems

n/a	Involved in the study
☒	☐ Antibodies
☒	☐ Eukaryotic cell lines
☒	☐ Palaeontology and archaeology
☐	☒ Animals and other organisms
☒	☐ Clinical data
☒	☐ Dual use research of concern
☒	☐ Plants

Methods

n/a	Involved in the study
☒	☐ ChIP-seq
☒	☐ Flow cytometry
☒	☐ MRI-based neuroimaging

Animals and other research organisms

Policy information about studies involving animals; ARRIVE guidelines recommended for reporting animal research, and Sex and Gender in Research

Laboratory animals	No laboratory animals were used in this study
Wild animals	Apis Mellifera
Reporting on sex	female (sterile worker bees)
Field-collected samples	n/a
Ethics oversight	No Ethical approval was required for this study

Note that full information on the approval of the study protocol must also be provided in the manuscript.

Plants

Seed stocks	*Report on the source of all seed stocks or other plant material used. If applicable, state the seed stock centre and catalogue number. If plant specimens were collected from the field, describe the collection location, date and sampling procedures.*
Novel plant genotypes	*Describe the methods by which all novel plant genotypes were produced. This includes those generated by transgenic approaches, gene editing, chemical/radiation-based mutagenesis and hybridization. For transgenic lines, describe the transformation method, the number of independent lines analyzed and the generation upon which experiments were performed. For gene-edited lines, describe the editor used, the endogenous sequence targeted for editing, the targeting guide RNA sequence (if applicable) and how the editor was applied.*
Authentication	*Describe any authentication procedures for each seed stock used or novel genotype generated. Describe any experiments used to assess the effect of a mutation and, where applicable, how potential secondary effects (e.g. second site T-DNA insertions, mosiacism, off-target gene editing) were examined.*
